# Morphologic and Genetic Characterization of Ilheus Virus, a Potential Emergent Flavivirus in the Americas

**DOI:** 10.3390/v15010195

**Published:** 2023-01-10

**Authors:** Jessica A. Plante, Kenneth S. Plante, Vsevolod L. Popov, Divya P. Shinde, Steven G. Widen, Michaela Buenemann, Mauricio L. Nogueira, Nikos Vasilakis

**Affiliations:** 1 Department of Microbiology and Immunology, University of Texas Medical Branch, Galveston, TX 77555-0609, USA; 2 World Reference Center for Emerging Viruses and Arboviruses, University of Texas Medical Branch, Galveston, TX 77555-0609, USA; 3 Institute for Human Infection and Immunity, University of Texas Medical Branch, Galveston, TX 77555-0610, USA; 4 Department of Pathology, University of Texas Medical Branch, Galveston, TX 77555-0609, USA; 5 Department of Biochemistry and Molecular Biology, University of Texas Medical Branch, Galveston, TX 77555-0679, USA; 6 Department of Geography and Environmental Studies, New Mexico State University, Las Cruces, NM 88003-8801, USA; 7 Center for Vector-Borne and Zoonotic Diseases, University of Texas Medical Branch, Galveston, TX 77555-0609, USA; 8 Department of Dermatological, Infectious and Parasitic Diseases, Faculdade de Medicina de São José do Rio Preto 15090-000, SP, Brazil

**Keywords:** Ilheus virus, ILHV, flavivirus, morphologic and genetic characterization

## Abstract

Ilheus virus (ILHV) is a mosquito-borne flavivirus circulating throughout Central and South America and the Caribbean. It has been detected in several mosquito genera including *Aedes* and *Culex*, and birds are thought to be its primary amplifying and reservoir host. Here, we describe the genomic and morphologic characterization of ten ILHV strains. Our analyses revealed a high conservation of both the 5′- and 3′-untranslated regions but considerable divergence within the open reading frame. We also showed that ILHV displays a typical flavivirus structural and genomic organization. Our work lays the foundation for subsequent ILHV studies to better understand its transmission cycles, pathogenicity, and emergence potential.

## 1. Introduction

**Figure 1:**
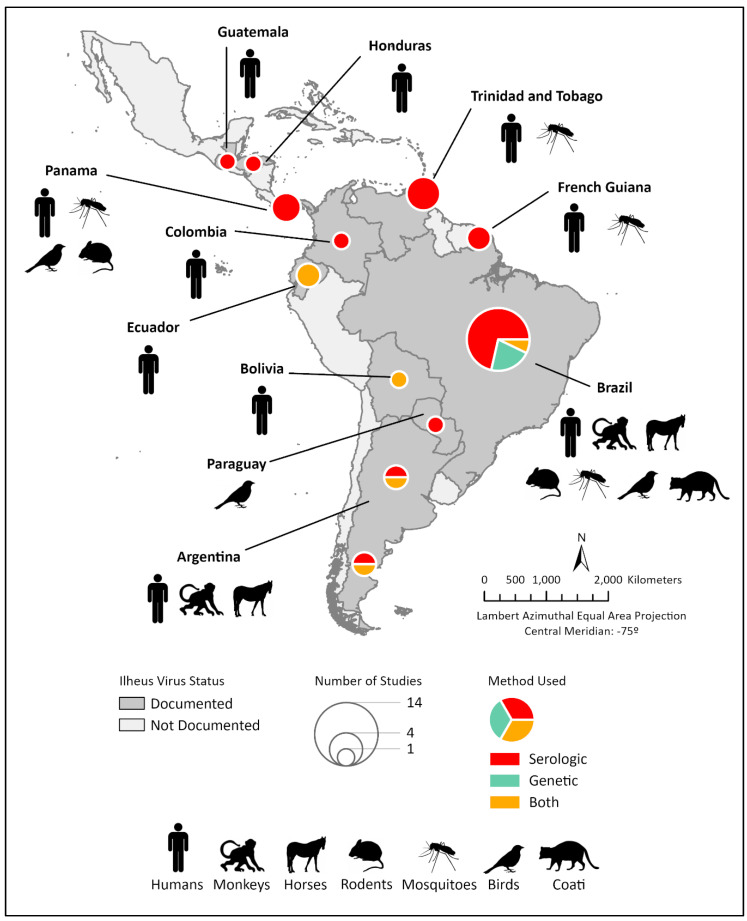
Geographic range and epidemiological landscape of Ilheus virus. Countries with evidence of ILHV circulation are named and indicated by dark grey shading. Hosts from which Ilheus virus and/or antibody have been identified within a given country are indicated by representative graphic(s). Pie charts within a given country indicate the number of studies identifying ILHV by size and the method of identification by color.

Ilheus virus (ILHV) is a mosquito-borne flavivirus (family *Flaviviridae*, genus *Flavivirus*) first isolated from *Aedes* and *Psorophora* spp. mosquitoes during an epidemiological investigation of yellow fever in the city of Ilheus, Bahia State, Brazil, in 1944 [1,2]. ILHV is maintained in an enzootic transmission cycle between birds and arboreal mosquitoes (*Aedes*, *Culex*, *Coquillettidia*, *Haemagogus*, *Sabethes*, *Trichoprosopon*, *Psorophora* and *Ochlerotatus*) [3,4,5,6,7,8,9]. Since its initial isolation, ILHV has been primarily isolated or detected in arboreal mosquitoes, birds, and humans throughout Central America, the Caribbean (Trinidad and Tobago) and South America [6,7,8,9,10,11,12,13,14,15,16,17] (Figure 1). However, several serological surveys have demonstrated the presence of ILHV antibodies in a wide range of vertebrates, including rodents [4,18,19], coatis [16], tortoises [4], water buffalo [20], bats [21], birds [4,16,17,18,22,23,24], horses [25,26,27,28], sloths [19,29,30], monkeys [16,18,29,31,32,33,34,35], and humans [1,10,18,36,37,38,39,40,41,42,43,44,45], sampled in both sylvatic, rural and urban ecotypes, thus suggesting a broader geographic and vertebrate host range of transmission (reviewed in [46]).

The handful of complete ILHV genome sequences available in public databases (GenBank accession numbers KC481679.1, NC_009028.2, AY632539.4, MK332106.1, and MH932545.1) indicate a typical flavivirus genome structure: a single-stranded RNA genome of ca. 10.7 kb in length encoding three structural proteins (C, prm/M, and E) and seven non-structural proteins (NS1, NS2A, NS2B, NS3, NS4A, NS4B, and NS5) flanked by 5′ and 3′ untranslated regions. Here, we leveraged the extensive virus collection of the World Reference Center for Emerging Viruses and Arboviruses (WRCEVA) to morphologically and genetically characterize several archived ILHV strains representing over six decades of sampling throughout South America.

## 2. Materials and Methods

### 2.1. Cells and Viruses

African green monkey kidney cells (CCL-81, hereafter referred to as Vero) and *Aedes albopictus* C6/36 cells were purchased from American Type Culture Collection (ATCC, Bethesda, MD, USA). Vero cells were maintained in Dulbecco’s Modified Eagle’s Medium (ThermoFisher Scientific, Waltham, MA, USA) supplemented with 5% (*vol*/*vol*) heat-inactivated fetal bovine serum (Atlanta Biologicals, Flowery Branch, GA, USA) and 1% (*vol*/*vol*) penicillin–streptomycin (ThermoFisher Scientific; 100 U/mL and 100 μg/mL, respectively) in a humidified 37 °C incubator with 5% CO_2_. C6/36 cells were maintained in an equal mixture of Minimum Essential Medium (ThermoFisher Scientific) and Leibovitz’s L-15 medium (ThermoFisher Scientific) supplemented with 10% (*vol*/*vol*) heat-inactivated fetal bovine serum, 5% (*vol*/*vol*) tryptose phosphate broth (ThermoFisher Scientific), 1% (*vol*/*vol*) non-essential amino acids (ThermoFisher Scientific), a 7.5% solution of 0.5% (*vol*/*vol*) sodium bicarbonate (ThermoFisher Scientific), 1 mM L-glutamine (ThermoFisher Scientific), and 1% (*vol*/*vol*) penicillin–streptomycin (100 U/mL and 100 μg/mL, respectively) in a humidified 28 °C incubator with 5% CO_2_. The 331, FSE 0800, H 2944, Original, PE 163615, PE 20545, ZPC 659, ZPC 804, ZCM 228, and BeH 7445 Ilheus strains (Table 1) were received as lyophilized stocks (World Reference Center for Emerging Viruses and Arboviruses, UTMB, Galveston, TX, USA). Of note, strain H 2944 is a higher passaged derivative of strain PE 20545.

### 2.2. Extraction of Viral RNA

Viral RNA from 140 µL of a cell culture supernatant was extracted using the QIAmp RNA mini kit (Qiagen, Hilden, Germany) and resuspended in 50 μL of RNase/DNase and protease-free water (Ambion, Austin, TX, USA).

### 2.3. Next-Generation Sequencing

Next-generation sequencing was performed on stocks of the passage histories described in Table 1. Viral RNA (~0.9 µg) was fragmented by incubation at 94 °C for eight minutes in 19.5 µL of a fragmentation buffer (Illumina, San Diego, CA, USA). A sequencing library was prepared from the sample RNA using a TruSeq RNA v2 (Illumina) kit following the manufacturer’s protocol. Samples were sequenced on a HiSeq 1500 (Illumina) using the 2 × 50 paired-end protocol, except for ZCM 228 and BeH 7445, which were sequenced on a NextSeq 550 (Illumina) in the paired-end 75 base format. Reads in the fastq format were quality-filtered, and any adapter sequences were removed using Trimmomatic software [47]. The de novo assembly program ABySS [48] was used to assemble the reads into contigs using several different sets of reads and k values from 20 to 40. In all samples, host reads were filtered out before de novo assembly. The longest contigs were selected, and reads were mapped back to the contigs using bowtie2 [49] and visualized with the Integrated Genomics Viewer [50] to verify that the assembled contigs were correct. A total of 17.4, 12.7, 13.0, 13.7, 9.9, 9.2, 13.3, 16.0, 10.3, and 9.4 million read pairs were generated for the samples containing the FSE 0800, Original, PE 20545, H 2944, PE 163615, ZPC 659, ZPC 804, 331, ZCM 228, and BeH 7445 ILHV strains, respectively. Read pairs mapping to the virus in each sample comprised ~1.2 million (6.75%), 572,000 (4.5%), 1.86 million (14.5%), ~175,000 (1.3%), 779,000 (7.9%), 1.9 million (20.6%), 286,000 (2.2%), 1.2 million (7.7%), 401,000 (3.9%), and 3.95 million (42.3%), respectively.

### 2.4. Rapid Amplification of cDNA Ends (RACE)

The genomic termini for the Original, 331, FSE 0800, PE 20545, and ZCM 228 ILHV strains were determined using the FirstChoice RLM RACE kit (Invitrogen, Vilnius, Lithuania). RACE was performed on the ZCM 228 strain of the passage history described in Table 1. RACE was performed on the Original, 331, FSE 0800, and PE 20545 strains of the passage histories described in Table 1 plus one additional passage in Vero cells. Self-ligated RNAs were amplified using GoTaq (Promega, Madison, WI, USA) with the virus-specific primers Ilheus_3_Outer (5′-AAGTGTGGAACAGGGTCTGG-3′) in the forward orientation and Ilheus_5_Outer (5′-CTCTCCGTGGTGAGGAATGT-3′) in the reverse orientation. The approximately 1400 nt amplicons were purified using the Zymoclean gel DNA recovery kit (Zymo Research, Irvine, CA, USA) prior to sequencing with the Ilheus_3_Inner (5′-CTGGGTTACCAAAGCCGTTA-3′) primer in the forward orientation and the Ilheus_5_Inner (5′-GCATGGTGGTCAGTTCCTTT-3′) primer in the reverse orientation. Sanger dideoxy sequencing was performed using the BigDye Terminator v3.1 Cycle Sequencing kit (Applied Biosystems, Austin, TX, USA) and a 3500 Genetic Analyzer machine (Applied Biosystems, Austin, TX, USA).

### 2.5. Transmission Electron Microscopy

For ultrastructural analysis, Vero and C6/36 cells infected for 3 days with the Original, H 2944, and ZPC 804 strains (passage histories in Table 1) were fixed for at least 1 h in a mixture of 2.5% formaldehyde prepared from paraformaldehyde powder and 0.1% glutaraldehyde in a 0.05 M cacodylate buffer (pH 7.3), to which 0.03% picric acid and 0.03% CaCl_2_ were added. The monolayers were washed in a 0.1 M cacodylate buffer, and cells were scraped off and further processed as pellets. The pellets were postfixed in 1% OsO_4_ in a 0.1 M cacodylate buffer (pH 7.3) for 1 h, washed with distilled water, and en-bloc stained with 2% aqueous uranyl acetate for 20 min at 60 °C. The pellets were dehydrated in ethanol, processed through propylene oxide, and embedded in Poly/Bed 812 (Polysciences, Warrington, PA, USA). Ultrathin sections were cut on a Leica EM UC7 ultramicrotome (Leica Microsystems, Buffalo Grove, IL, USA), stained with lead citrate, and examined with a JEM-1400 (JEOL USA, Inc., Peabody, MA, USA) transmission electron microscope at 80 kV.

### 2.6. Genome Annotation

RNA structure prediction was performed in mFold [51], and manual annotations were added with Biorender.com. The 5′ UTR structures were trimmed to include the entire 5′ UTR and the first 17 nucleotides of the capsid gene to complete the stem–loop B (SLB) structure. The 3′ UTR structures were trimmed immediately following the TAA stop codon, and mFold was restricted to an 80-nucleotide maximum distance between paired bases. Repeat sequences in the 3′ UTR were identified using Unipro UGENE v41.0 [52]. Transmembrane domains (TMDs) were predicted on the basis of their alignment with TMDs identified in other flaviviruses [53,54,55,56,57,58,59,60]. Protein cleavage sites were identified by their alignment with the previously deposited and annotated ILHV Original GenBank sequence NC_009028.2 [61], as well as with analysis with SignalP 4.1 [62]. N-linked glycosylation sites were predicted with NetNGlyc 1.0 [63]; sites with a jury agreement of 9/9 are reported unless otherwise noted. Phosphorylation sites were predicted with NetPhos 3.1 [64,65]; sites with a score of ≥0.8 are reported. Cysteine bridge formation was predicted on the basis of alignment with cysteine bridges that have been experimentally verified to exist in other flaviviruses. Glycosylation, phosphorylation, and cysteine bridge prediction was performed with the Original strain of ILHV. The polyprotein was visualized with Biorender.com. To compare sequence elements across several mosquito-borne flavivirus species, the Original ILHV strain was aligned with sequences from GenBank: West Nile virus (WNV) NY99 (accession AF196835.2) [66], Japanese encephalitis virus (JEV) Nakayama (accession EF571853.1), Rocio virus (ROCV) SPH 34675 (accession MF461639.1) [67], dengue 1 virus (DENV1) WestPac (accession U88535.1) [68], dengue 2 virus (DENV2) New Guinea C (accession KM204118.1) [69], dengue 3 virus (DENV3) H87 (accession KU050695.1) [69], dengue 4 virus (DENV4) H241 (accession KR011349.2) [69], yellow fever virus (YFV) Asibi (accession KF769016.1) [70], and Zika virus (ZIKV) MR 766 (accession HQ234498.1) [71]. Alignments were performed with Clustal Omega in MegAlign Pro v17.2.1 (DNASTAR, Madison, WI, USA), and manual annotations were added with Biorender.com.

### 2.7. Nucleotide Sequence Accession Numbers

The following ILHV genome sequences were determined in this study: 331, FSE 0800, Original, PE 163615, PE 20545, H 2944, ZPC 659, ZPC 804, ZCM 228, and BeH 7445, with accession numbers OP947882–OP947891, respectively (Table 2).

### 2.8. Phylogenetic Analysis

The evolutionary history was inferred by using the maximum likelihood method and the general time reversible model [72]. The tree with the highest log likelihood (−29,698.16) is shown. Initial tree(s) for the heuristic search were automatically obtained by applying Neighbor-Join and BioNJ algorithms to a matrix of pairwise distances estimated using the maximum composite likelihood (MCL) approach and then selecting the topology with the superior log likelihood value. A discrete Gamma distribution was used to model the evolutionary rate differences among sites (5 categories (+*G*, parameter = 0.9858)). The rate variation model allowed for some sites to be evolutionarily invariable ([+*I*], 57.03% sites). The tree was drawn to scale, with branch lengths measured in the number of substitutions per site. This analysis involved 14 nucleotide sequences (13 ILHV and Rocio virus (ROCV) as the outgroup used to root the tree). The included codon positions were 1st + 2nd + 3rd + Noncoding. There were a total of 10,278 positions in the final dataset. Evolutionary analyses were conducted in MEGA11 [73,74].

## 3. Results

### 3.1. Virus Morphology

**Figure 2:**
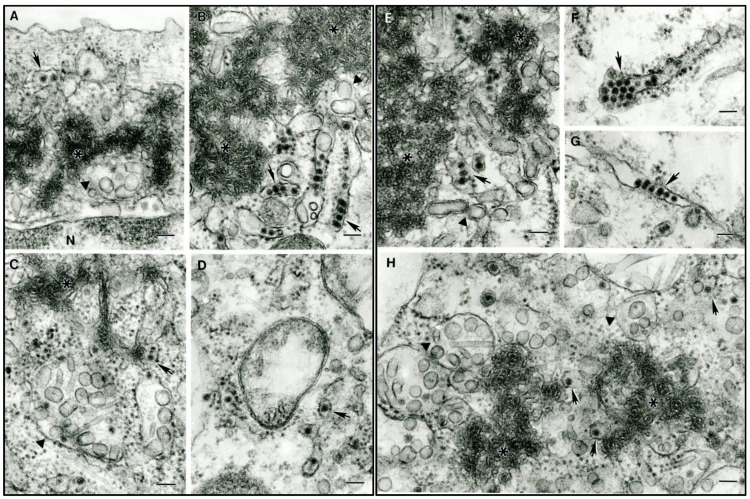
Ultrastructure of ILHV in Vero and C6/36 cells. Arrows show virus particles inside granular endoplasmic reticulum cisterns. Asterisks show convoluted membranes, and solid triangles show smooth membrane structures (SMS). (**A**) Original strain in Vero cells. (**B**) H 2944 strain in Vero cells. (**C**,**D**) H 2944 strain in C6/36 cells. (**E**–**G**) ZPC 804 strain in Vero cells. (**H**) ZPC 804 strain in C6/36 cells. Bar—100 nm.

In ultrathin sections of infected Vero and C6/36 cells, different structures related to the flavivirus replication cycle were consistently observed (Figure 2A–H)—convoluted membranes (asterisks in Figure 2A–C,E), smooth membrane structures (SMS) within endoplasmic reticulum cisterns (solid triangles in Figure 2A–C,E,H) that are considered viral RNA processing sites, and immature virus particles within the cisterns of granular endoplasmic reticulum (Figure 2A–H, solid arrows). Virus particles were ~45 nm in diameter, and all the described structures of ILHV were typical for the genus *Flavivirus.*

### 3.2. Phylogenetic Analysis

**Figure 3:**
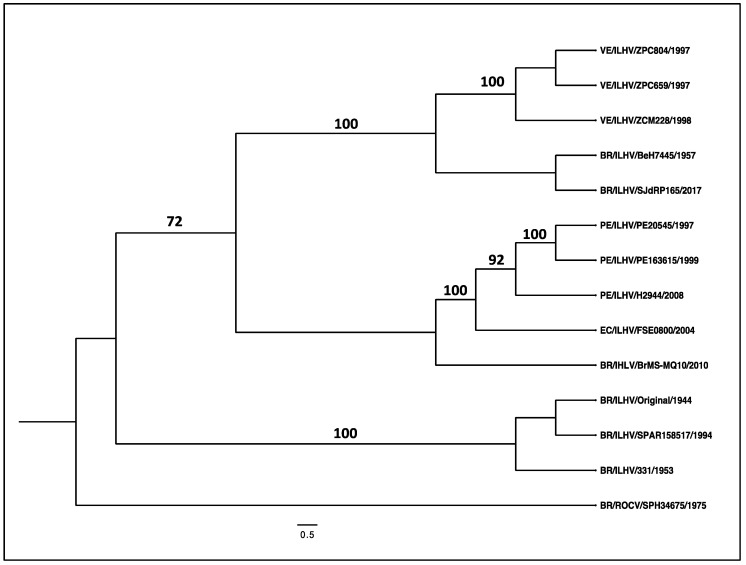
Phylogeny analysis of ILHV. The phylogeny is based on complete ORFs of the ILHV and Rocio virus using a total of 14 sequences. Evolutionary analysis was conducted in MEGA version 11 and the tree produced in version 1.4.4 of FigTree. The scale shows a genetic distance of 0.5 for nucleotide sequence divergence. Numbers indicate bootstrap values for groups to the right. Sequences are labeled by country of origin (BR = Brazil; EC = Ecuador; PE = Peru; and VE = Venezuela), followed by viral species, followed by viral strain, followed by year of isolation.

We provided the first phylogenetic analysis of ILHV strains based on 13 complete open reading frame sequences, 10 of which were determined with the NGS leveraging of the resources of WRCEVA. The strains were isolated between 1944 and 2017 (mostly in Brazil, Venezuela, Ecuador and Peru), representing strains from diverse localities throughout South America, including ROCV used as an outgroup to root the ILHV tree (Figure 3). While there are approximately 30 ILHV nucleotide sequences in GenBank, most represent partial NS5 gene sequences, thus limiting the ability of comprehensive analyses to obtain insights into ILHV’s phylogeography and spatiotemporal dynamics of transmission. Despite the limited number of complete ORF sequences available at our disposal, the analysis revealed considerable genetic diversity reflecting ILHV’s continual divergence and diverse geographic distribution (Figure 3).

### 3.3. Genomic Characterization

The ILHV genome comprises approximately 10.8 kilobases (kb) of a single-stranded RNA of positive polarity. A single open reading frame (ORF) of 10,278 nucleotides (Table 2) is flanked by untranslated regions (UTRs) at the 5′ and 3′ ends. The 5′ UTR is 92–93 nt long and is capped with a type I 5′ cap, while the 387–388 nt 3′ UTR lacks the classical polyadenylation site [75,76].

#### 3.3.1. 5′ and 3′ UTRs

**Figure 4:**
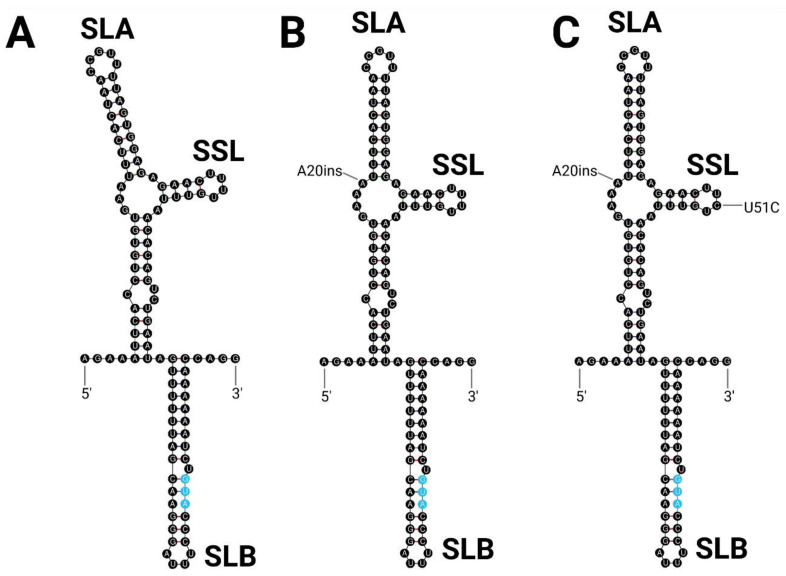
5′ UTR Structure of ILHV. Predicted 5′ UTR structures of (**A**) Original and 331; (**B**) FSE 0800, PE 163615, PE 20545, H 2944, and BeH 7445; and (**C**) ZPC 659, ZPC 804, and ZCM 228 strains of ILHV. The conserved flavivirus structural domains stem–loop A (SLA), stem–loop B (SLB), and the side stem–loop (SSL) are labeled. Unique sequence features are indicated with text. The ORF start codon is indicated in blue.

The 5′ UTR consists of 93 nucleotides and is highly conserved, with only two points of variation between strains. The two oldest strains, Original and 331, have two adenines beginning at nucleotide 18 in comparison with the three adenines observed in all other strains (Table 2). The three Venezuelan strains were found to have a cytosine at nucleotide 51, while all other ILHV strains were found to have a uracil at the equivalent position. Both of these changes were found to be located in loop structures and to have minimal impact on the free energy of the predicted structure of the 5′ UTR (Figure 4).

**Figure 5:**
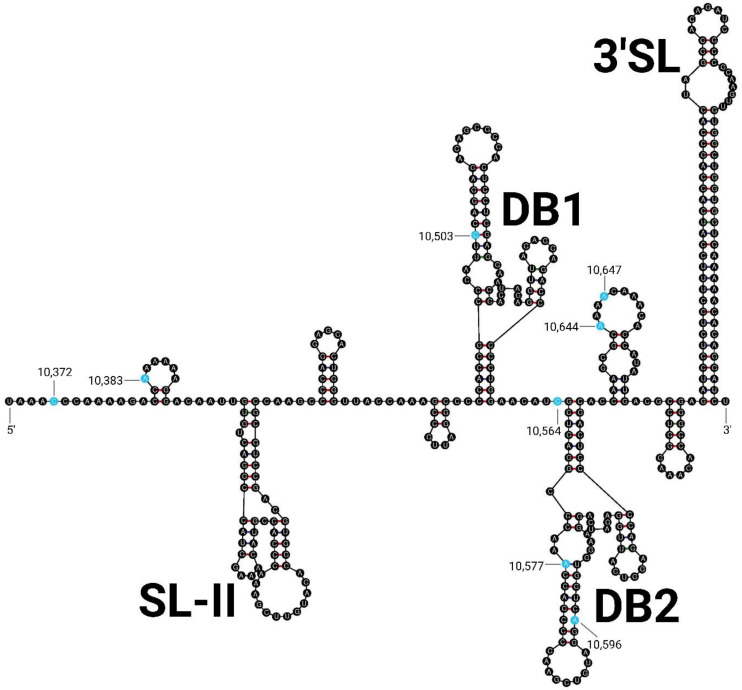
3′ UTR Structure of ILHV. Predicted structure of the consensus ILHV 3′ UTR. The conserved flavivirus structural domains stem–loop II (SL-II), dumbbell 1 (DB1), dumbbell 2 (DB2), and 3′ stem–loop (3′SL) are labeled. Variable sequence features are highlighted in blue, and their position in the ILHV Original sequence is indicated.

The 3′ UTR of ILHV is 387–388 nucleotides long and was predicted to contain the SL-II, DB1, DB2, and 3′ SL structural elements (Figure 5) [77]. The 3′ UTR of ILHV also contains the conserved sequence 1 (CS1), conserved sequence 2 (CS2), conserved sequence 3 (CS3), and repeated CS2 (RCS2) elements of the flavivirus 3′ UTR but lacks the repeated CS3 (RCS3) element characteristic of the JEV group and the YF-R1, YF-R2, and YF-R3 elements characteristic of YFV [78,79]. Of the total 387–388 nucleotides, only eight were found to vary between strains. Two variable positions were shown to be unique to the oldest strains, 331 and Original: C10,503U and A10,644G, with the nucleotides numbered according to their positions in the Original genome here and for all remaining descriptions of variable nucleotides. One variable position was found to be unique to the H 2944, PE 20545, and PE 163615 Peruvian isolates: A10,596G. Three variable positions were found to be unique to the Venezuelan isolates ZPC 659, ZPC 804, and ZCM 228: C10,372U, A10,383del, and A10,577G. One variable position was found to be unique to the three Venezuelan isolates and the FSE 0800 Ecuadorian isolate: C10,564U. The A10,647G variable position was found to be unique to strain ZCM 228. Strain BeH 7455 matched the consensus at all eight variable positions of the 3′ UTR. The predicted initial free energy ranged from −136.0 kcal/mol for the Original and 331 strains to −145.3 kcal/mol for the Peruvian isolates.

#### 3.3.2. Open Reading Frame

**Figure 6:**
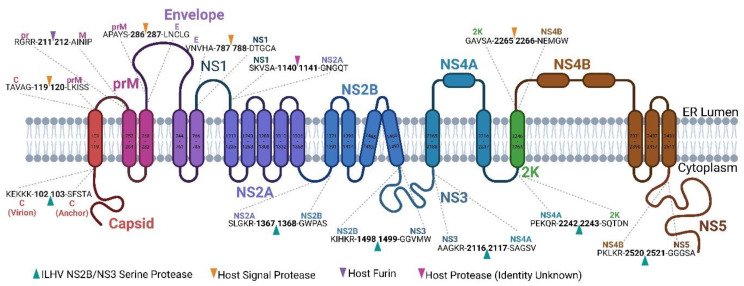
Structure and cleavage of ILHV polyprotein. Schematic map of the ILHV polyprotein. Proteins, putative protein cleavage sites and the corresponding proteases, and transmembrane domains are annotated. Amino acid numbering corresponds to the position in ILHV Original.

The ILHV open reading frame (ORF) was found to be 3425 amino acids long, with 99.3–100% sequence identity (Table 2, Supplementary Figure S1). The resulting polyprotein encompasses the three structural proteins (capsid (C), pre-membrane/mature membrane (prM/M), and envelope (E)) toward its amino terminus and the seven nonstructural proteins (NS1, NS2A, NS2B, NS3, NS4A, NS4B, and NS5) toward its carboxy terminus (Figure 6). In line with previously characterized flaviviruses, cleavage was predicted to be achieved by a combination of host and viral proteases [80].

##### Capsid

The ILHV capsid (C) protein was found to be 119 amino acids long, with the 102 amino-terminal residues forming the virion C protein and the 17 carboxy-terminal residues forming a trans-membrane domain (TMD) that anchors the C to the membrane. The C protein contains five sites that were shown to vary between ILHV strains (Supplementary Figure S1). Residue 13 is a threonine in all but ZPC 804, in which it is an alanine, and residue 33 is a leucine in all but Original and 331, in which it is a phenylalanine. The remaining variable residues (106, 114, and 116) were found to be located within the anchor portion of C. Interestingly, residue N72 of the ILHV C was predicted to be glycosylated. The glycosylation of the C protein has not been reported for other flaviviruses; however, comparable in silico prediction does hint at possible N72 glycosylation in ROCV (Supplementary Figure S2), albeit with reduced confidence. Seven serine residues (2, 22, 24, 38, 70, 93, and 103) and one threonine (74) were predicted to be phosphorylated. All predicted phosphorylation sites except S103 were shown to be located in the cytoplasmic region of C; S103 is located immediately to the carboxy side of the predicted NS3 cleavage site and is the first residue of the anchor portion of C.

##### prM

The precursor membrane (prM) protein of ILHV was found to be 167 amino acids long. Cleavage by the host furin protease separates the 92 amino-terminal amino acids that comprise the ‘precursor’ component of prM from the 75 carboxy-terminal amino acids that comprise the mature M component. Three positions in prM were shown to vary between strains: the three oldest strains (Original, 331, and BeH 7445) have an aspartic acid at position 8 where all other strains have a glycine, the three Venezuelan strains (ZPC 659, ZPC 804, and ZCM 228) have an isoleucine at position 87 where all other strains have a valine, and H 2944 has an alanine at position 90 where all other strains have a glycine (Supplementary Figure S1). The N15 residue of ILHV prM was predicted in silico to be glycosylated. This residue is known to be glycosylated in WNV and JEV, and it has been associated with receptor usage, tropism, virion assembly, and pathogenesis [81,82,83]. Other flaviviruses, such as DENV, YFV, and Zika virus (ZIKV), are also known to have glycosylated prM proteins [84,85,86]. Three serine residues (5, 27, and 102), four threonine residues (49, 109, 114, and 117), and three tyrosine residues (51, 75, and 134) were predicted to be phosphorylated. Half of the predicted phosphorylation sites, S109, T109, T114, T117, and Y134, were found to be located within the M portion of prM; of these, all but Y134 were found to be located in the predicted ectodomain of M. Three disulfide bridges have been experimentally verified in the crystal structure of DENV-2 [87]; all six cysteines are conserved across all nine flaviviruses considered here and were predicted to form disulfide bridges between C34–C69, C45–C81, and C53–C67 of ILHV (Supplementary Figure S2).

##### Envelope

The ILHV envelope (E) protein was found to be 501 amino acids long and to contain six variable residues (Supplementary Figure S1). Three points of variation only occur in a single strain: residue 157 is a glycine in BeH 7455 and an alanine in all other strains, residue 306 is an arginine in ZCM 228 and a lysine in all other strains, and residue 388 is an arginine in strain 331 and a glutamine in all other strains. The other three points varied by strain origin: residue 147 is an isoleucine in the two oldest strains (Original and 331) and a threonine in all other strains, residue 367 is a lysine in the three oldest strains (Original, 331, and BeH 7455) and an asparagine in all other strains, and residue 390 is a serine in the Peruvian strains (H 2944, PE 2054, and PE 163615) and an asparagine in all other strains. The N154 residue of the ILHV E was predicted to be glycosylated in silico. This glycosylation site is widespread among flaviviruses but is not universal at either the genus or species level. It has been shown to modulate neuroinvasion, receptor binding, particle assembly, mosquito midgut invasion, and immunogenicity [82,83,88,89,90,91]. Ten serine residues (68, 69, 95, 232, 238, 364, 365, 368, 402, and 482), seven threonine residues (55, 115, 126, 251, 314, 318, and 350), and three tyrosine residues (96, 176, and 329) were predicted to be phosphorylated. Six disulfide bridges have been experimentally verified in WNV [92] and DENV-2 [93]; all twelve cysteines were found to be conserved across all nine flaviviruses considered here and were predicted to form disulfide bridges between C3–C80, C60–C121, C74–C105, C92–C116, C190–C288, and C305–C336 of ILHV (Supplementary Figure S2).

##### NS1

NS1, a non-structural protein often linked to RNA replication and immune modulation, exists as intracellular monomers and dimers and is secreted as a hexamer [94,95]. The ILHV NS1 was found to be 353 amino acids long and to contain six variable residues (Supplementary Figure S1). Three of the variable residues are found exclusively in Venezuelan strains: residue 54 is an isoleucine in ZCM 228 and a valine in all other strains, residue 146 is a leucine in ZPC 659 and ZPC 804 and a serine in all other strains, and residue 245 is a threonine in ZCM 228, ZPC 659, and ZPC 804 and an isoleucine in all other strains. The Peruvian strains (H 2944, PE 20545, and PE 163615) contain an arginine at residue 261 where all other strains contain a lysine. The two oldest strains (331 and Original) contain a glutamic acid at residue 328 where all other strains contain an aspartic acid. Strain BeH 7455 contains a serine at residue 293 where all other strains contain a glycine. No N-linked glycosylation sites were predicted with high confidence in silico; the N207 site, which is highly conserved among flaviviruses and has been linked to pathogenicity, was predicted in silico not to be glycosylated [96,97]. Eight serine residues (49, 140, 178, 204, 209, 252, 298, and 305) and four threonine residues (38, 105, 165, and 303) were predicted to be phosphorylated. Multiple lines of evidence have confirmed the six disulfide bonds of flavivirus NS1. Crystallography has been used to identify the cysteine pairs of WNV and DENV-2 as 1–2, 3–4, 5–6, 7–12, 8–9, and 10–11 [98]. These results were in slight contrast to the previously reported DENV-2 arrangement of 1–2, 3–4, 5–6, 7–12, 8–10, and 9–11, the last two pairs being determined with tandem mass spectrometry [99]. This experimental discrepancy was likely caused by the difficulty in resolving the ninth, tenth, and eleventh cysteines due to their proximity in the primary structure of NS1 (CCKNC in ILHV and ROCV and CCRSC in other mosquito-borne flaviviruses [98]). All twelve cysteines were found to be conserved across all nine flaviviruses considered here and were predicted to form disulfide bridges between C4–C15, C55–C143, C179–C223, C281–C330, and either C229–C313 and C314–C317 or C229–C314 and C313–C317 of ILHV (Supplementary Figure S2).

##### NS2A

The flavivirus NS2A protein binds the 3′ UTR during RNA replication and is necessary for particle assembly [59,100,101]. In ILHV, the NS2A protein was found to be 227 amino acids long and to contain three variable residues: residue 24 is an arginine in the three Brazilian strains (331, Original, and BeH 7455) and a lysine in all other strains, residue 72 is an isoleucine in ZCM 228 and a valine in all other strains, and residue 204 is an alanine in the two oldest strains (331 and Original) and a valine in all other strains (Supplementary Figure S1). No asparagines were predicted with a high degree of confidence in silico to be glycosylated. Phosphorylation was predicted at two serine residues (70 and 189) and two threonine residues (29 and 92). There are no conserved cysteine residues in the NS2A protein of flaviviruses (Supplementary Figure S2), and disulfide bonds within NS2A have not been reported or experimentally verified in other flaviviruses.

##### NS2B

NS2B is an essential co-factor for the NS3 protease [102,103]. The ILHV NS2B protein was found to be 131 amino acids long, with only a single point of variation: residue 60 is a valine in the three Venezuelan strains (ZCM 228, ZPC 659, and ZPC 804) and an isoleucine in all other strains (Supplementary Figure S1). No glycosylation sites were predicted in the ILHV NS2B, nor have any been reported for other flaviviruses. Three serine residues (61, 72, and 81) were predicted to be phosphorylated, as was the threonine at residue 125. There are no conserved cysteine residues in the NS2B protein of flaviviruses (Supplementary Figure S2), and disulfide bonds within NS2B have not been reported or experimentally verified in other flaviviruses.

##### NS3

The flavivirus NS3 protein is critical to viral replication, serving as both a serine protease to cleave the viral polyprotein and an RNA helicase and RNA triphosphatase for genomic replication [104,105,106]. In ILHV, NS3 was found to be 618 amino acids long and to be highly conserved. Only a single strain, ZPC 804, was found to vary at a single residue, possessing a valine at residue 30 where all other strains possess an isoleucine (Supplementary Figure S1). Residue N66 was predicted in silico to be glycosylated; however, the presence of a proline at residue 67 in the N-X-S/T N-linked glycosylation motif renders this prediction questionable, and the glycosylation of the NS3 protein has not been reported in other flaviviruses. Phosphorylation was predicted at nine serine residues (71, 253, 302, 390, 393, 426, 468, 547, and 609), ten threonine residues (175, 180, 190, 245, 267, 272, 303, 318, 377, and 479), and two tyrosine residues (472 and 555). The cysteines at ILHV C262 and C563 were found to be fully conserved amongst the nine considered flaviviruses and C375 was found to be conserved among all but YFV (Supplementary Figure S2). However, the X-ray crystallography of the helicase portion of ILHV NS3, comprising residues 177–618, made no note of any disulfide bond formation [107].

##### NS4A and 2K

The flavivirus NS4A and 2K are membrane-bound proteins involved in membrane remodeling, the RNA replication complex, and NS3 protease activity [55,108,109,110]. The ILHV NS4A protein was found to be 149 amino acids long, from which the 23 carboxy-terminal amino acids are cleaved to form the 2K protein. The mature NS4A protein was only found to vary at a single position, residue 69, which is a threonine in strain 331 and an alanine in all other strains (Supplementary Figure S1). One additional residue, position 17 in 2K and position 143 in the immature NS4A protein, is a valine in the Peruvian strains (H 2944, PE 20545, and PE 163615) and a leucine in all other strains. Glycosylation was not predicted, nor has it been reported for other flaviviruses. Only a single phosphorylation site was predicted: a tyrosine at residue 15 of NS4A. There are no conserved cysteine residues in the NS4A or 2K proteins of flaviviruses (Supplementary Figure S2), and disulfide bonds have not been reported or experimentally verified in other flaviviruses.

##### NS4B

Flavivirus NS4B is a component of the RNA replication complex and suppresses several host immune responses [111,112,113,114]. The ILHV NS4B protein was found to be 255 amino acids long with six variable residues (Supplementary Figure S1). Three of those variable residues are unique to the Venezuelan strains (ZCM 228, ZPC 659, and ZPC 804): residue 15 is an arginine in the Venezuelan strains and a lysine in the other strains, residue 22 is a histidine in the Venezuelan strains and an aspartic acid in the other strains, and residue 83 is an asparagine in the Venezuelan strains and a serine in the other strains. Two residues were found to be unique to the Peruvian strains (H 2944, PE 20545, and PE 163615) and the single Ecuadorian strain (FSE 0800): residue 20 is a threonine in these four strains and a serine in the other strains, and residue 30 is a histidine in these four strains and a glutamine in the other strains. Residue 164 is an isoleucine in ZPC 804 and a threonine in all other strains. Glycosylation was not predicted for ILHV NS4B. The N219 residue was identified as a potential glycosylation site with weak confidence by in silico analysis; however, the equivalent position in DENV-2 was also predicted to be glycosylated in silico but was experimentally demonstrated to not be glycosylated [114,115]. Phosphorylation is predicted at two serine residues (19 and 20) and at two threonine residues (8 and 9). ILHV possesses three cysteine residues in its NS4B protein: C102, C182, and C227. Although none of those cysteines are universally conserved between the nine considered flaviviruses (Supplementary Figure S2), two cysteines in equivalent positions (C99 and C178) of DENV showed chemical shifts in an NMR analysis suggestive of possible disulfide bond formation [116].

##### NS5

The NS5 protein of flaviviruses is critical to RNA replication, with both RNA-dependent RNA polymerase and RNA guanylyltransferase activity [117,118,119]. The ILHV NS5 protein was found to be 905 amino acids long and to vary at 11 residues (Supplementary Figure S1). The two oldest strains (331 and Original) were found to vary at four of these residues: residue 72 is an arginine in the oldest strains and a lysine in the other strains, residue 567 is a lysine in the oldest strains and a glutamine in the other strains, residue 619 is an alanine in the oldest strains and a valine in the other strains, and residue 886 is a cysteine in the oldest strains and a tyrosine in the other strains. Residue 843 was found to vary in the three oldest strains (331, Original, and BeH 7455), which possess an isoleucine where the other strains possess a leucine. Residue 424 is an isoleucine in the Peruvian strains (H 2944, PE 20545, and PE 163615) and an arginine in the other strains. Strain ZPC 659 was found to vary at two unique positions: residues 200 and 546 are both threonines in ZPC 659 and alanines in the other strains. Strain FSE 0800 was found to vary at two unique positions: residue 790 is an asparagine in FSE 0800 and an aspartic acid in the other strains, and residue 827 is a tyrosine in FSE 0800 and a histidine in the other strains. Residue 206 is an isoleucine in strain BeH 7445 and a valine in the other strains. Residue N213 may be glycosylated according to in silico analysis; however, the prediction had only weak confidence and glycosylation has not been reported for NS5 in other flaviviruses. Eighteen serines (46, 128, 153, 214, 271, 320, 389, 500, 504, 524, 596, 640, 660, 665, 745, 748, 751, and 836), ten threonines (59, 93, 161, 396, 422, 544, 573, 695, 794, and 895), and one tyrosine (883) were predicted to be phosphorylated. The phosphorylation of NS5 has been associated with binding to NS3 in the replication complex [120]. The ILHV NS5 protein possesses nineteen cysteine residues, eight of which (C395 C448, C451, C669, C713, C732, C757, and C784) were found to be conserved in all nine flaviviruses considered here (Supplementary Figure S2). However, disulfide bridges have not been noted in the crystal structures of full-length NS5 from DENV2, DENV3, JEV or Zika virus despite the presence of 10–16 cysteines in these proteins [121,122,123,124,125].

## 4. Discussion

Despite first being characterized in 1946 [1,2], ILHV remains an understudied virus. Severe and fatal human disease associated with ILHV infection is sporadic [11,12,13,14,15,34,46,126,127,128], and there have been no known epidemic or epizootic outbreaks of ILHV. However, the introduction of WNV to the United States in 1999 [66] and ZIKV to Brazil in 2015 [129] both demonstrated that flaviviruses are capable of rapid expansion in previously naïve regions and that that circulation can be associated with significant disease and potentially emergent pathologies [130,131]. Given the frequency with which ILHV is found by either the isolation or detection of viral RNA in mosquitoes, birds, humans, and other potential host species [3,4,5,6,7,8,9,10,11,12,13,14,15,16,17], as well as the prevalence of ILHV-reactive antibodies in serological surveys [1,4,10,16,17,18,19,20,21,22,23,24,25,26,27,28,29,30,31,32,33,34,35,36,37,38,39,40,41,42,43,44,45], it is worth considering ILHV circulation’s epidemic potential. It is therefore prudent to characterize ILHV now, so that the initial knowledge and tools are in place should ILHV ever emerge as a more widespread threat to human health.

The ten new full-length genomes deposited into GenBank as part of this characterization contribute a significant increase in the ILHV sequence diversity available for analysis. Furthermore, the temporal and geographic spread of these strains makes them a particularly valuable addition. The genomic organization, cellular organization, and structure of ILHV is typical of flaviviruses with no major deviations. However, there are a few features worth noting. The 3′ UTR of ILHV was predicted to possess the CS1, CS2, CS3 and RCS2 conserved sequence elements and to lack the RCS3, YF-R1, YF-R2, and YF-R3 conserved sequence elements. The presence of the CS3 element and the absence of the RCS3 element leaves ILHV straddling the JEV group, which contains both sequence elements, and the DENV group, which contains neither sequence element [132]. The potential glycosylation of residue N72 in the capsid protein is also noteworthy in light of the similar prediction for ROCV and the lack of glycosylation reported in the capsid protein of other flaviviruses. It should be emphasized that our genomic characterization of ILHV relied on in silico predictions and must be experimentally verified (e.g., crystal structure). However, this work lays the foundation for the future study of ILHV and emphasizes the unique role of collections such as the WRCEVA in characterizing understudied pathogens with potential for emergence.

## Figures and Tables

**Table 1:** History of Ilheus strains utilized in this study.

Strain	Host	Country	Year	Passage History *
Original	*Aedes* and *Psorophora* spp.	Brazil	1944	SM-29, Vero-1
331	N/A	Brazil	1953	SM-2
BeH 7445	Human	Brazil	1957	SM-?, Vero-1
FSE 0800	Human	Ecuador	2004	Vero-3
H 2944	*Psorophora (Jan.) ferox*	Peru	1997	Vero-4, Hamster-1
PE 20545	*Psorophora (Jan.) ferox*	Peru	1997	Vero-3
PE 163615	*Culex (Cul.) coronator*	Peru	1999	Vero-3
ZPC 659	*Mesocricetus auratus*	Venezuela	1997	Vero-1
ZPC 804	*Mesocricetus auratus*	Venezuela	1997	C6/36-2
ZCM 228	*Psorophora (Jan.) ferox*	Venezuela	1998	C6/36-2

* SM—suckling mouse; C6/36—*Ae. albopictus* cell line; Vero—*Cercopithecus aethiops* (kidney epithelial cells); hamster—*Mesocricetus auratus.*

**Table 2:** Characterization of ILHV genetic sequences.

ILHV Strain ^a^	GenBank Accession	Genome Length (nt)	% Identity (nt) ^b^	% Identity (aa) ^b^	ORF (nt)	5′ UTR (nt)	3′ UTR (nt)
**Original**	OP947886	10,758	100.00	100.00	10,278	92	388
**331**	OP947882	10,758	99.95	99.94	10,278	92	388
**BeH 7445**	OP947885	10,758	96.65	99.65	10,278	93	387
**FSE 0800**	OP947883	10,759	95.49	99.47	10,278	93	388
**H 2944**	OP947884	10,759	95.71	99.42	10,278	93	388
**PE 20545**	OP947887	10,759	95.73	99.45	10,278	93	388
**PE 163615**	OP947888	10,759	95.73	99.45	10,278	93	388
**ZPC 659**	OP947890	10,759	95.40	99.36	10,278	93	388
**ZPC 804**	OP947891	10,758	95.41	99.33	10,278	93	387
**ZCM 228**	OP947889	10,758	95.40	99.36	10,278	93	387

^a^ Low passage ILHV isolates were obtained from the UTMB World Reference Center for Emerging Viruses and Arboviruses. ^b^ Denotes % identity compared to the Original reference strain.

## Data Availability

The ILHV genome sequences determined in this study have been deposited in the GenBank database with accession numbers OP947882–OP947891.

## Supplementary Materials

The following supporting information can be downloaded at: https://www.mdpi.com/article/10.3390/v15010195/s1, Figure S1: Alignment of ILHV Polyprotein; Figure S2: Alignment of Individual Proteins Across Multiple Flaviviruses.
