# Supplementary material for: Morphologic and Genetic Characterization of Ilheus Virus, a Potential Emergent Flavivirus in the Americas
Source: Viruses. 2023 Jan 10;15(1):195. doi: 10.3390/v15010195 (PMC9866216; doi:10.3390/v15010195)
Supplement: Supplementary file 1 [file viruses-15-00195-s001.zip › Supplementary Figure S2 - Flavivirus Alignments.pdf]

Capsid

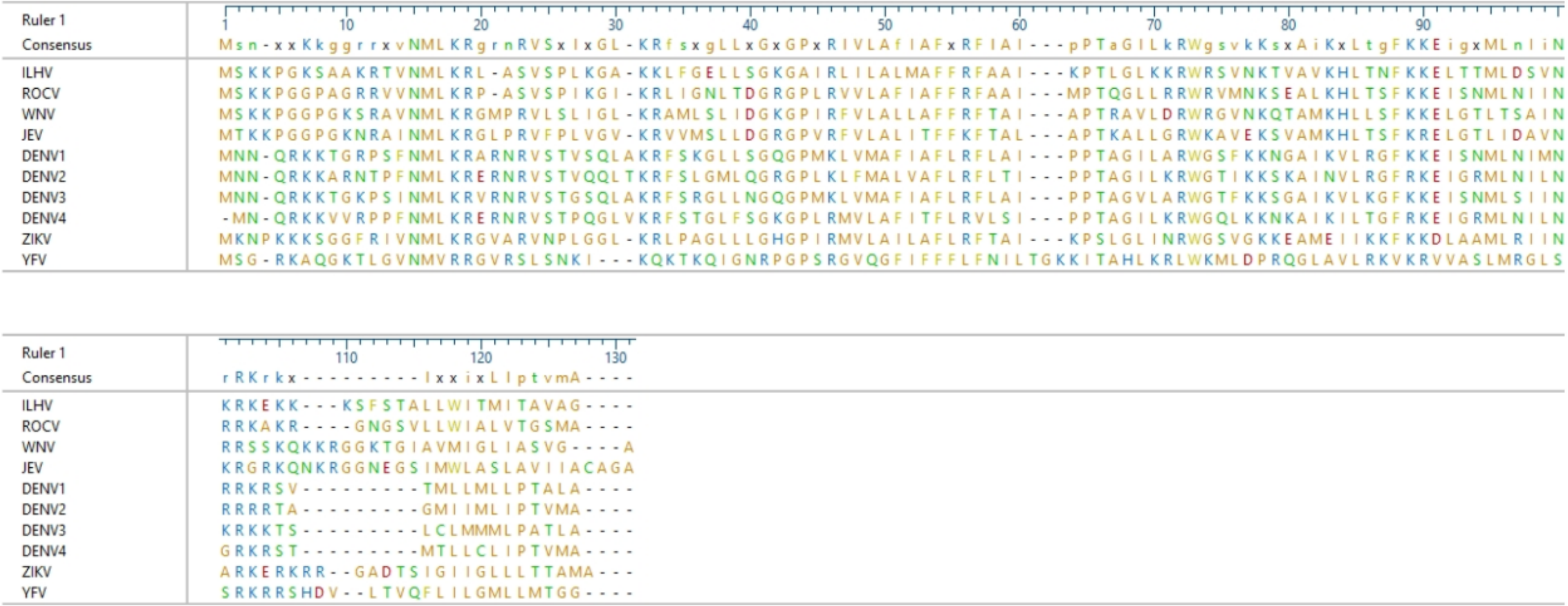

Supplementary Figure S2: Alignment of Individual Proteins Across Multiple Flaviviruses. Individual proteins from representative flaviviruses were aligned using the Clustal Omega algorithm in MegAlign Pro v17.2.1 (DNASTAR, Madison, WI). Amino acids are colored by side chain chemistry: aromatic residues (F, W, and Y) are yellow, acidic residues (D and E) are red, basic residues (R, H, and K) are blue, nonpolar residues (A, G, I, L, M, P, and V) are orange, and polar residues (C, N, Q, S, and T) are green). Gaps are represented as dashes. Black rectangles designate cysteine residues predicted to form disulfide bridges.

prM

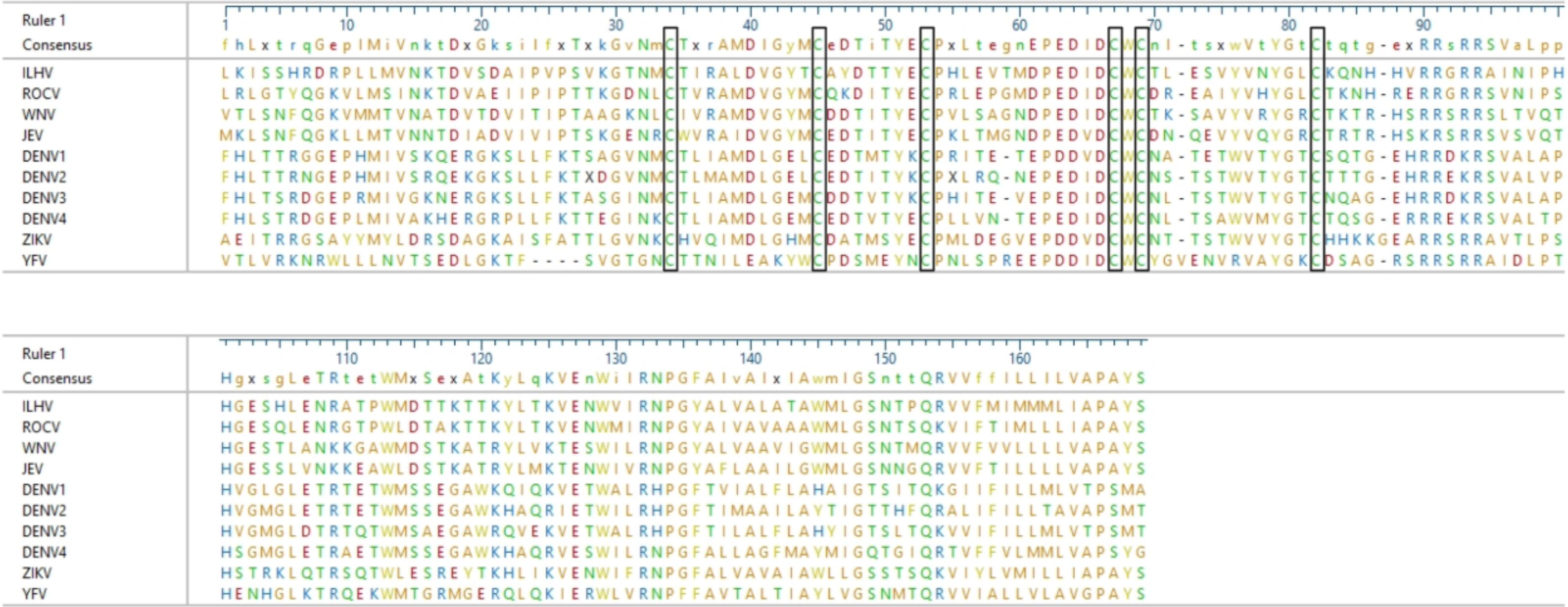

Supplementary Figure S2: Alignment of Individual Proteins Across Multiple Flaviviruses. Individual proteins from representative flaviviruses were aligned using the Clustal Omega algorithm in MegAlign Pro v17.2.1 (DNASTAR, Madison, WI). Amino acids are colored by side chain chemistry: aromatic residues (F, W, and Y) are yellow, acidic residues (D and E) are red, basic residues (R, H, and K) are blue, nonpolar residues (A, G, I, L, M, P, and V) are orange, and polar residues (C, N, Q, S, and T) are green). Gaps are represented as dashes. Black rectangles designate cysteine residues predicted to form disulfide bridges.

Envelope

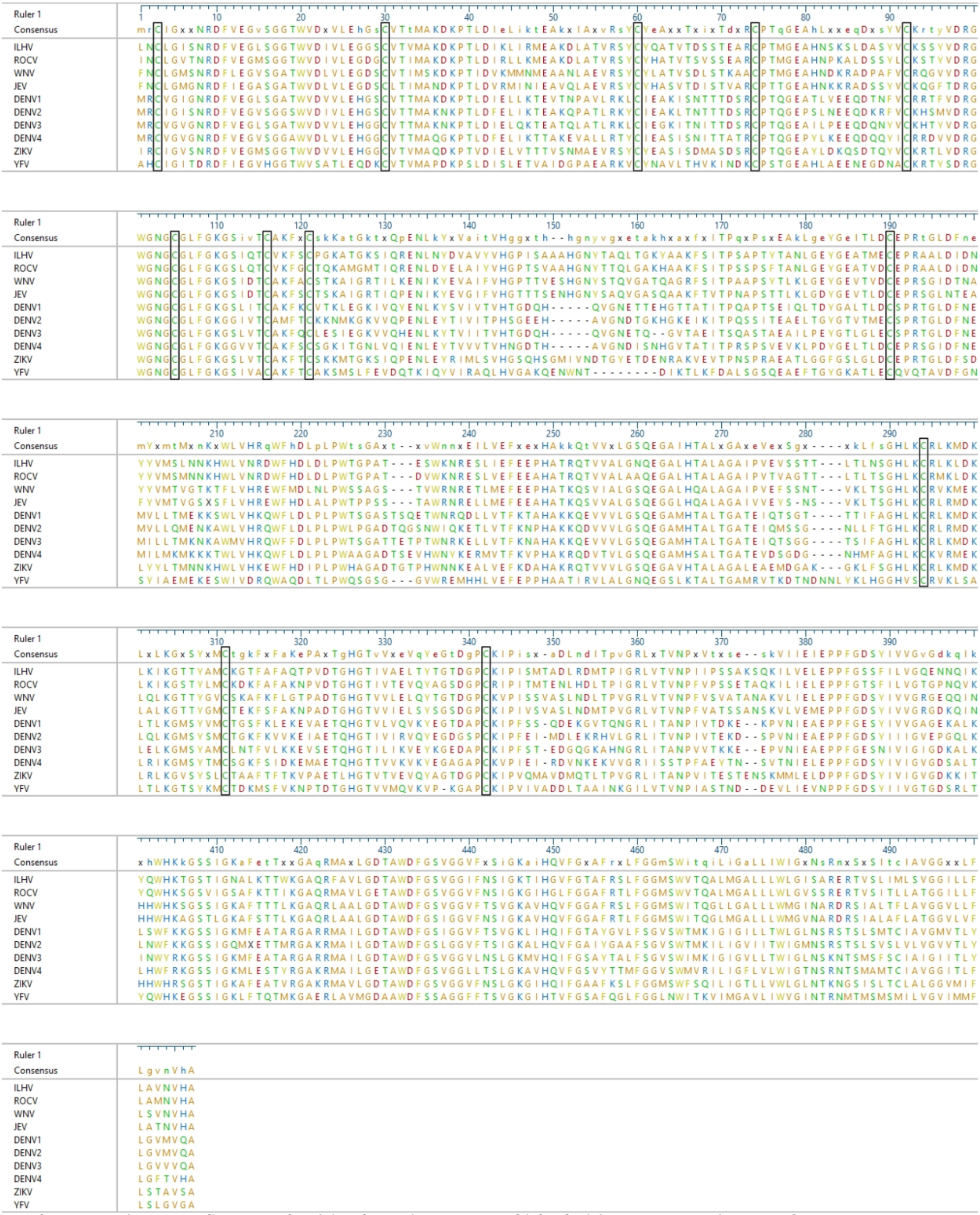

Supplementary Figure S2: Alignment of Individual Proteins Across Multiple Flaviviruses. Individual proteins from representative flaviviruses were aligned using the Clustal Omega algorithm in MegAlign Pro v17.2.1 (DNASTAR, Madison, WI). Amino acids are colored by side chain chemistry: aromatic residues (F, W, and Y) are yellow, acidic residues (D and E) are red, basic residues (R, H, and K) are blue, nonpolar residues (A, G, I, L, M, P, and V) are orange, and polar residues (C, N, Q, S, and T) are green). Gaps are represented as dashes. Black rectangles designate cysteine residues predicted to form disulfide bridges.

NS1

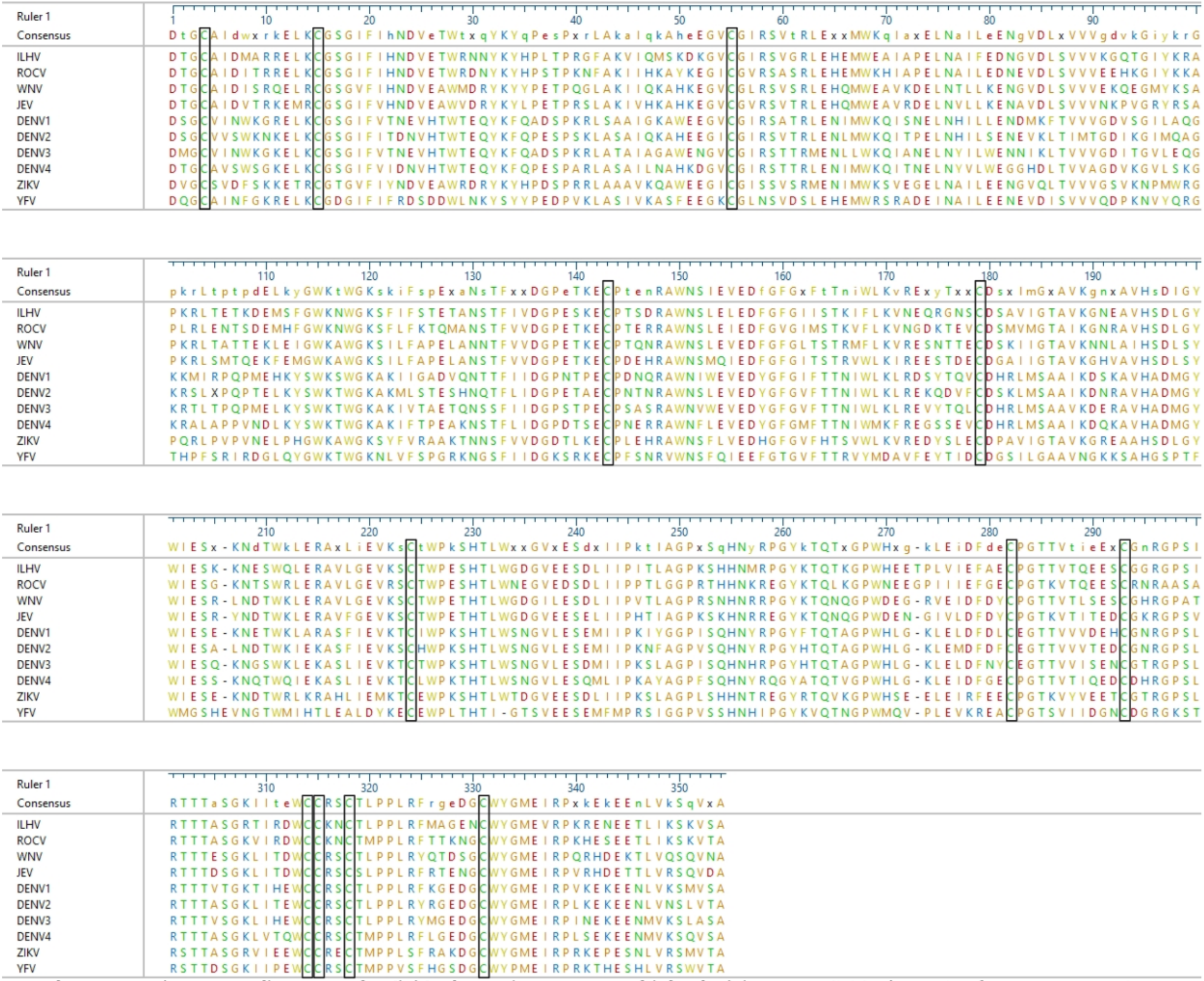

Supplementary Figure S2: Alignment of Individual Proteins Across Multiple Flaviviruses. Individual proteins from representative flaviviruses were aligned using the Clustal Omega algorithm in MegAlign Pro v17.2.1 (DNASTAR, Madison, WI). Amino acids are colored by side chain chemistry: aromatic residues (F, W, and Y) are yellow, acidic residues (D and E) are red, basic residues (R, H, and K) are blue, nonpolar residues (A, G, I, L, M, P, and V) are orange, and polar residues (C, N, Q, S, and T) are green). Gaps are represented as dashes. Black rectangles designate cysteine residues predicted to form disulfide bridges.

NS2A

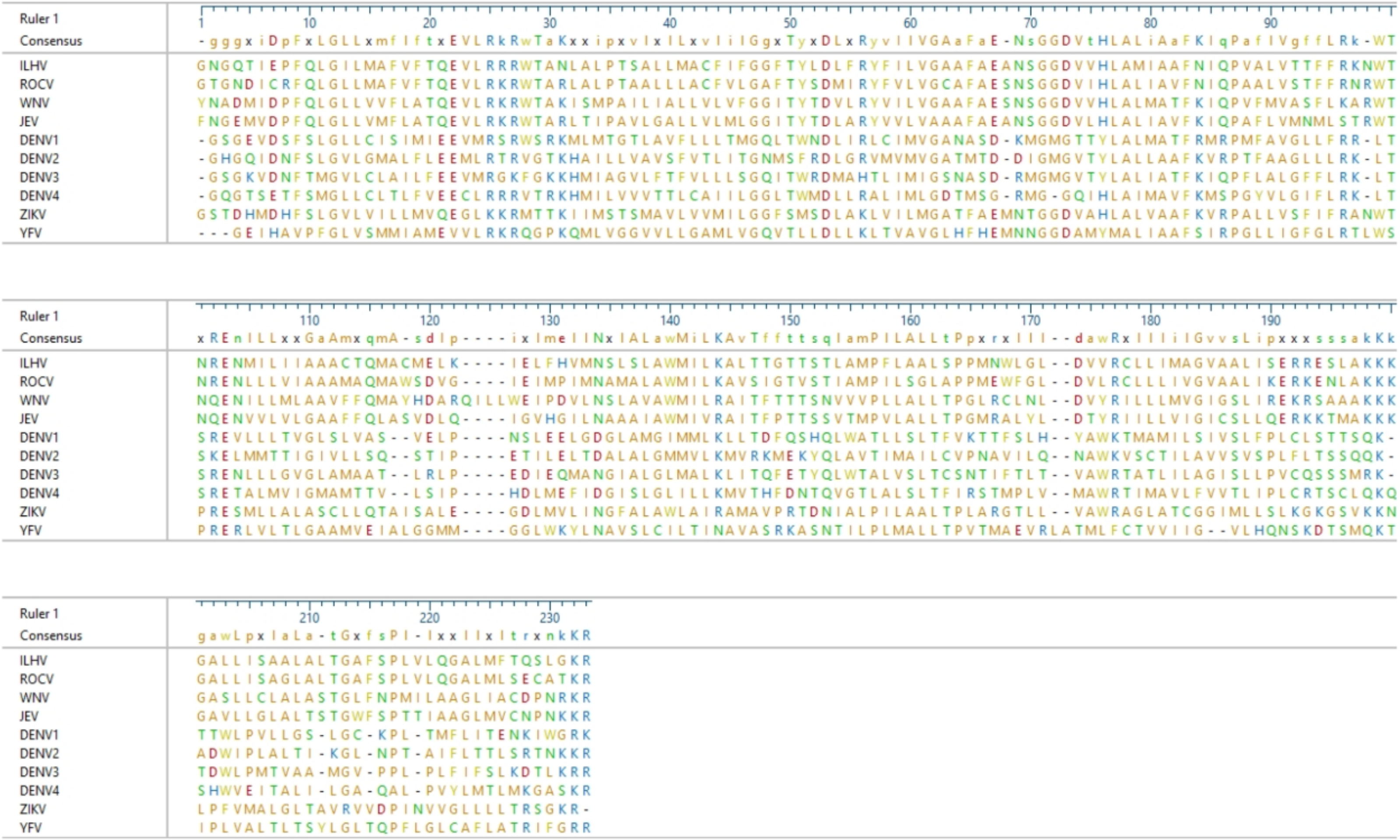

Supplementary Figure S2: Alignment of Individual Proteins Across Multiple Flaviviruses. Individual proteins from representative flaviviruses were aligned using the Clustal Omega algorithm in MegAlign Pro v17.2.1 (DNASTAR, Madison, WI). Amino acids are colored by side chain chemistry: aromatic residues (F, W, and Y) are yellow, acidic residues (D and E) are red, basic residues (R, H, and K) are blue, nonpolar residues (A, G, I, L, M, P, and V) are orange, and polar residues (C, N, Q, S, and T) are green). Gaps are represented as dashes. Black rectangles designate cysteine residues predicted to form disulfide bridges.

NS2B

```
Ruler 1    1        10        20        30        40        50        60        70        80        90
Consensus  SWPxnEvlxAVGLvsaLAGsLAkxDip-MAGPlaagGLLivxYVxSGkSxDxwLERAADVsWEeeAEITGsSpRLDVeLDdDGdFkxxxdegvPmltwll
ILHV       GWPASEVLTAVGMTFALAGSVARLDGGTMAIPLATMAILAVAYVLSGKSTDMWLERCADISWINEAEITGTSPRLDVELDSNGDFKMINDPGVPMWMWTC
ROCV       GWPASEVLTAIGMTIALAGSVARLDSGTMAIPLATTSILFVSYVLSGKSTDMWIERCADVTWEEEAEITGTSPRLDVELDDNGDFKMINDPGVPMWMWAS
WNV        GWPATEVMTAVGLMFAIVGGLAELDIDSMAIPMTIAGLMFAAFVISGKSTDMWIERTADISWESDAEITGSSERVDVRLDDDGNFQLMNDPGAPWKIWML
JEV        GWPATEFLSAVGLMFAIVGGLAELDIESMSIPFMLAGLMAVSYVVSGKATDMWLERAADISWEMDAAITGSSRRLDVKLDDDGDFHLIDDPGVPWKVWVL
DENV1      SWPLNEGIMAVGIVSILLSSLLKNDVP-LAGPLIAGGMLIACYVISGSSADLSLEKAAEVSWEEEAEHSGASHNILVEVQDDGTMKIKDEERDDTLTILL
DENV2      SWPLNEAIMAVGMVSILASSLLKNDIP-MTGPLVAGGLLTVCYVLTGRSADLELERAADVKWEDQAEISGSSPILSITISEDGSMSIKNEEEEQILTILI
DENV3      SWPLNEGVMAVGLVSILASSLLRNDVP-MAGPLVAGGLLIACYVITGTSADLTVEKAADVTWEEEAEQTGVSHNLMITVDDDGTMRIKDDETENILTVLL
DENV4      SWPLNEGIMAVGLVSLLGSALLKNDVP-LAGPMVAGGLLLAAYVMSGSSADLSLEKAANVQWDEMADITGSSPIIEVKQDEDGSFSIRDIEETNMITLLV
ZIKV       SWPPSEVLTAVGLICALAGGFAKADIE-MAGPMAAVGLLIVSYVVSGKSVDMYIERAGDITWEKDAEVTGNSPRLDVALDESGDFSLVEEDGPPMREIIL
YFV        SIPVNEALAAAGLVGVLAG-LAFQEMENFLGPIAVGGILMMLVSVAGRVDGLELKKLGEVSWEEEAEISGSSARYDVALSEQGEFKLLSEEKVPWDQVVM
```

```
Ruler 1            110       120       130
Consensus  rmgLlaisxxnPvaIPvtxagWxmxxKktkR
ILHV       RMGLMAMAAYNPVLIPVSMAGYWMTVKIHKR
ROCV       RMGLMCMAAYNPVLIPVSVAGYWMTRKIHKR
WNV        RMVCLAISAYTPWAILPSVVGFWITLQYTKR
JEV        RMSCIGLAALTPWAIVPAAFGYWLTLKTTKR
DENV1      KATLLAISGVYPMSIPATLFVWYFWQKKKQR
DENV2      RTGLLVISGLFPVSIPITAAAWYLWEVKKQR
DENV3      KTALLIVSGIFPYSIPATLLVWHTWQKQTQR
DENV4      KLALITVSGLYPLAIPVTMTLWYMWQVKTQR
ZIKV       KVVLMAICGMNPIAIPFAAGAWYVYVKTGKR
YFV        TSLALVGAAIHPFALLLVLAGWLFHVRGARR
```

Supplementary Figure S2: Alignment of Individual Proteins Across Multiple Flaviviruses. Individual proteins from representative flaviviruses were aligned using the Clustal Omega algorithm in MegAlign Pro v17.2.1 (DNASTAR, Madison, WI). Amino acids are colored by side chain chemistry: aromatic residues (F, W, and Y) are yellow, acidic residues (D and E) are red, basic residues (R, H, and K) are blue, nonpolar residues (A, G, I, L, M, P, and V) are orange, and polar residues (C, N, Q, S, and T) are green). Gaps are represented as dashes. Black rectangles designate cysteine residues predicted to form disulfide bridges.

NS3

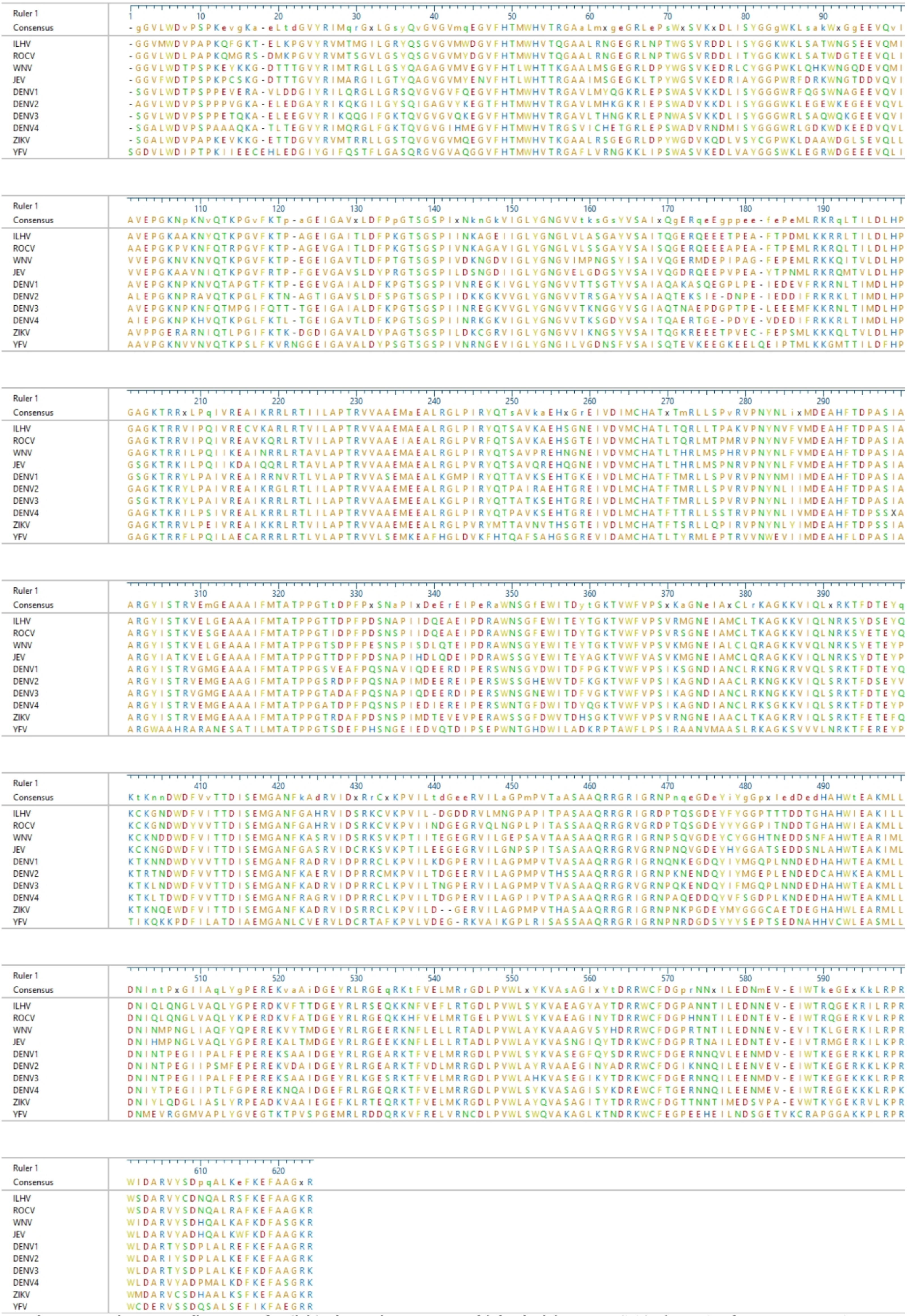

Supplementary Figure S2: Alignment of Individual Proteins Across Multiple Flaviviruses. Individual proteins from representative flaviviruses were aligned using the Clustal Omega algorithm in MegAlign Pro v17.2.1 (DNASTAR, Madison, WI). Amino acids are colored by side chain chemistry: aromatic residues (F, W, and Y) are yellow, acidic residues (D and E) are red, basic residues (R, H, and K) are blue, nonpolar residues (A, G, I, L, M, P, and V) are orange, and polar residues (C, N, Q, S, and T) are green). Gaps are represented as dashes. Black rectangles designate cysteine residues predicted to form disulfide bridges.

NS4A and 2K

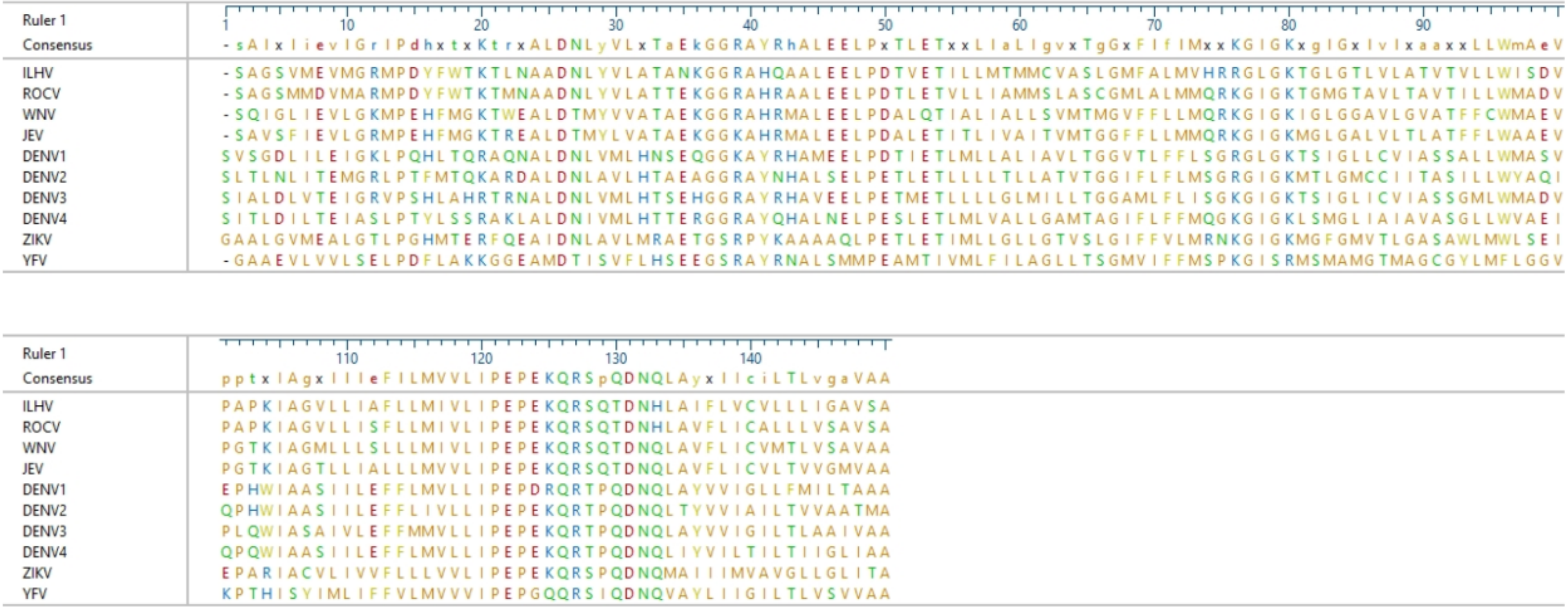

Supplementary Figure S2: Alignment of Individual Proteins Across Multiple Flaviviruses. Individual proteins from representative flaviviruses were aligned using the Clustal Omega algorithm in MegAlign Pro v17.2.1 (DNASTAR, Madison, WI). Amino acids are colored by side chain chemistry: aromatic residues (F, W, and Y) are yellow, acidic residues (D and E) are red, basic residues (R, H, and K) are blue, nonpolar residues (A, G, I, L, M, P, and V) are orange, and polar residues (C, N, Q, S, and T) are green). Gaps are represented as dashes. Black rectangles designate cysteine residues predicted to form disulfide bridges.

NS4B

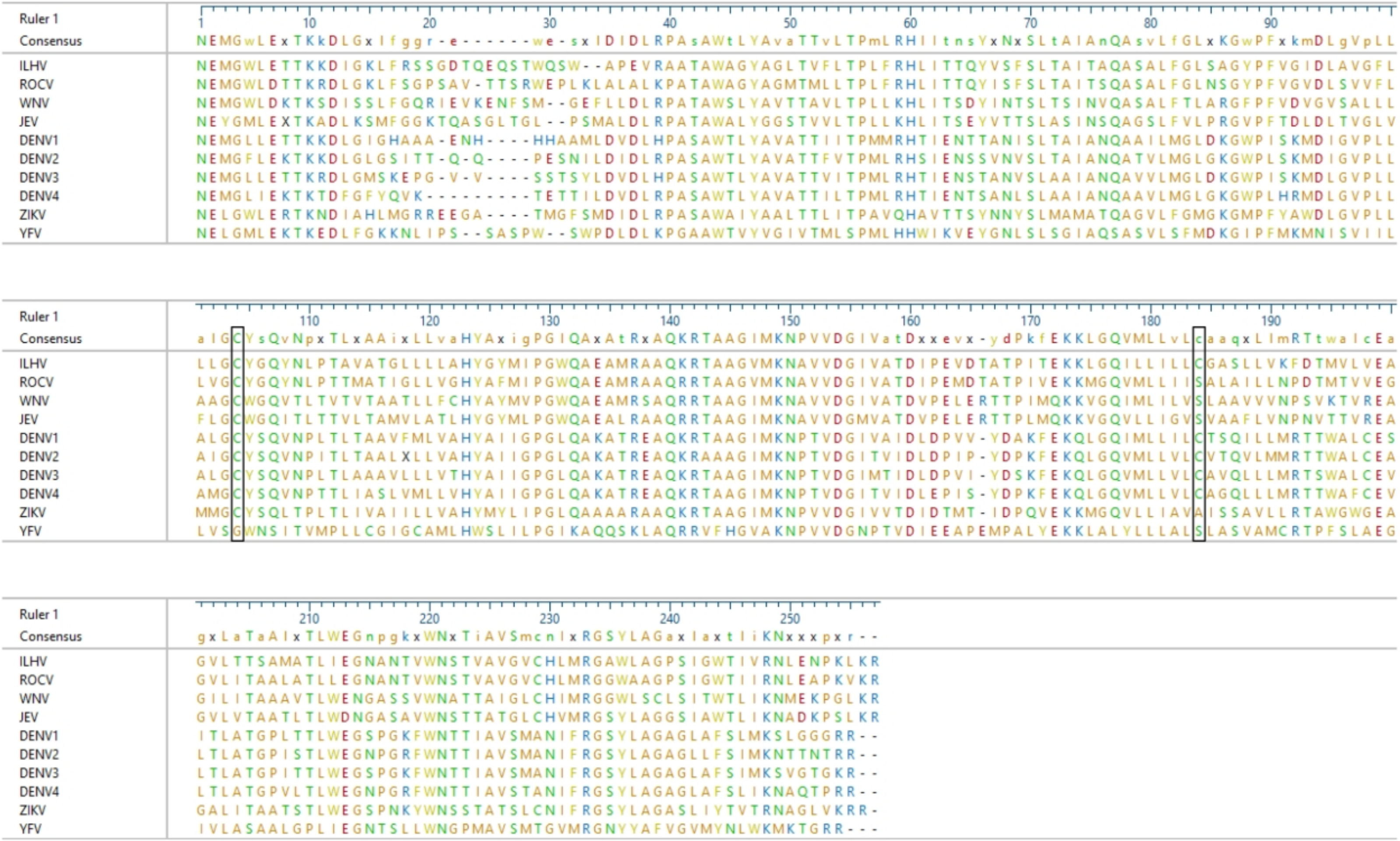

Supplementary Figure S2: Alignment of Individual Proteins Across Multiple Flaviviruses. Individual proteins from representative flaviviruses were aligned using the Clustal Omega algorithm in MegAlign Pro v17.2.1 (DNASTAR, Madison, WI). Amino acids are colored by side chain chemistry: aromatic residues (F, W, and Y) are yellow, acidic residues (D and E) are red, basic residues (R, H, and K) are blue, nonpolar residues (A, G, I, L, M, P, and V) are orange, and polar residues (C, N, Q, S, and T) are green). Gaps are represented as dashes. Black rectangles designate cysteine residues predicted to form disulfide bridges.

NS5

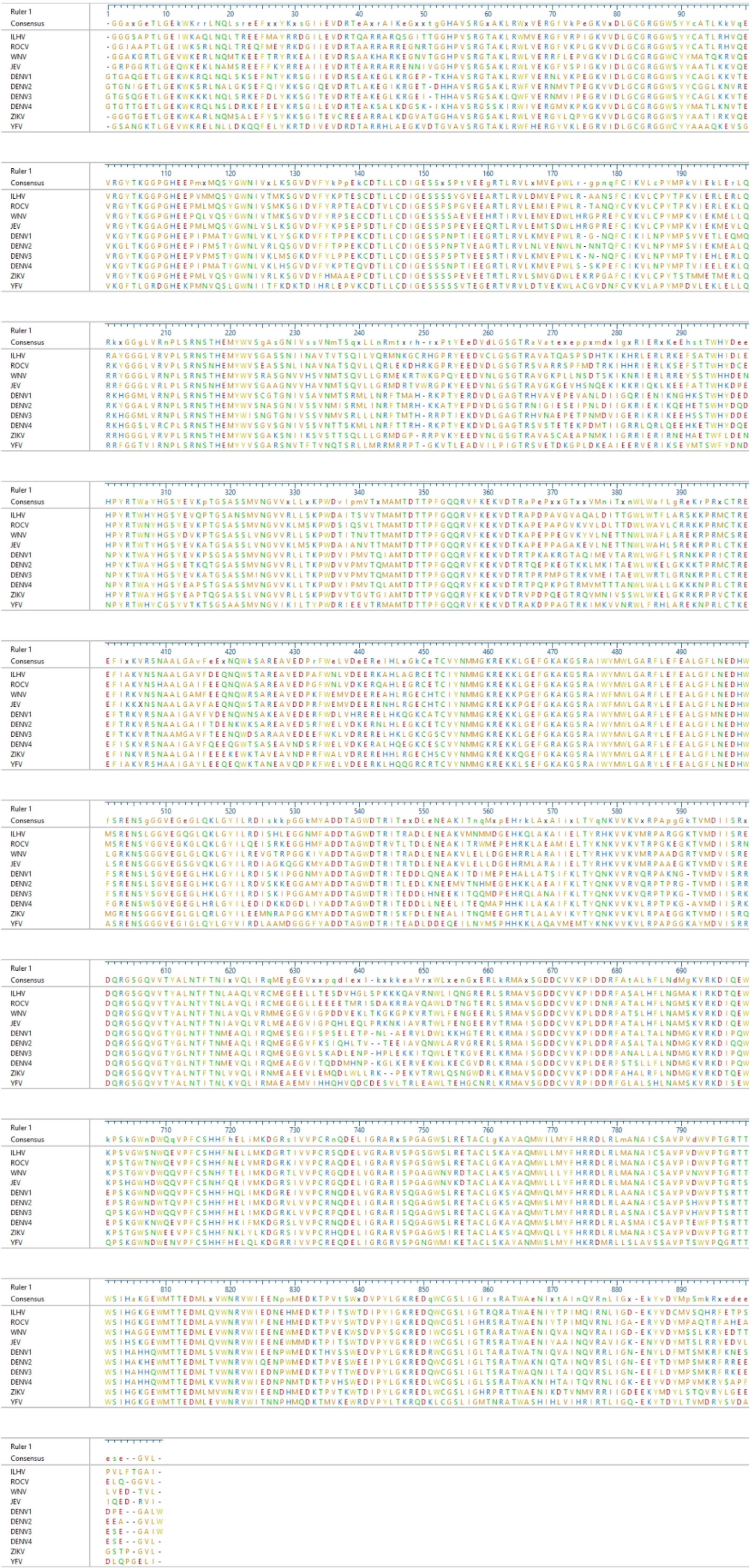

Supplementary Figure S2: Alignment of Individual Proteins Across Multiple Flaviviruses. Individual proteins from representative flaviviruses were aligned using the Clustal Omega algorithm in MegAlign Pro v17.2.1 (DNASTAR, Madison, WI). Amino acids are colored by side chain chemistry: aromatic residues (F, W, and Y) are yellow, acidic residues (D and E) are red, basic residues (R, H, and K) are blue, nonpolar residues (A, G, I, L, M, P, and V) are orange, and polar residues (C, N, Q, S, and T) are green). Gaps are represented as dashes. Black rectangles designate cysteine residues predicted to form disulfide bridges.
