# Supplementary figures and images for: Morphologic and Genetic Characterization of Ilheus Virus, a Potential Emergent Flavivirus in the Americas
Source: Viruses. 2023 Jan 10;15(1):195. doi: 10.3390/v15010195 (PMC9866216; doi:10.3390/v15010195)

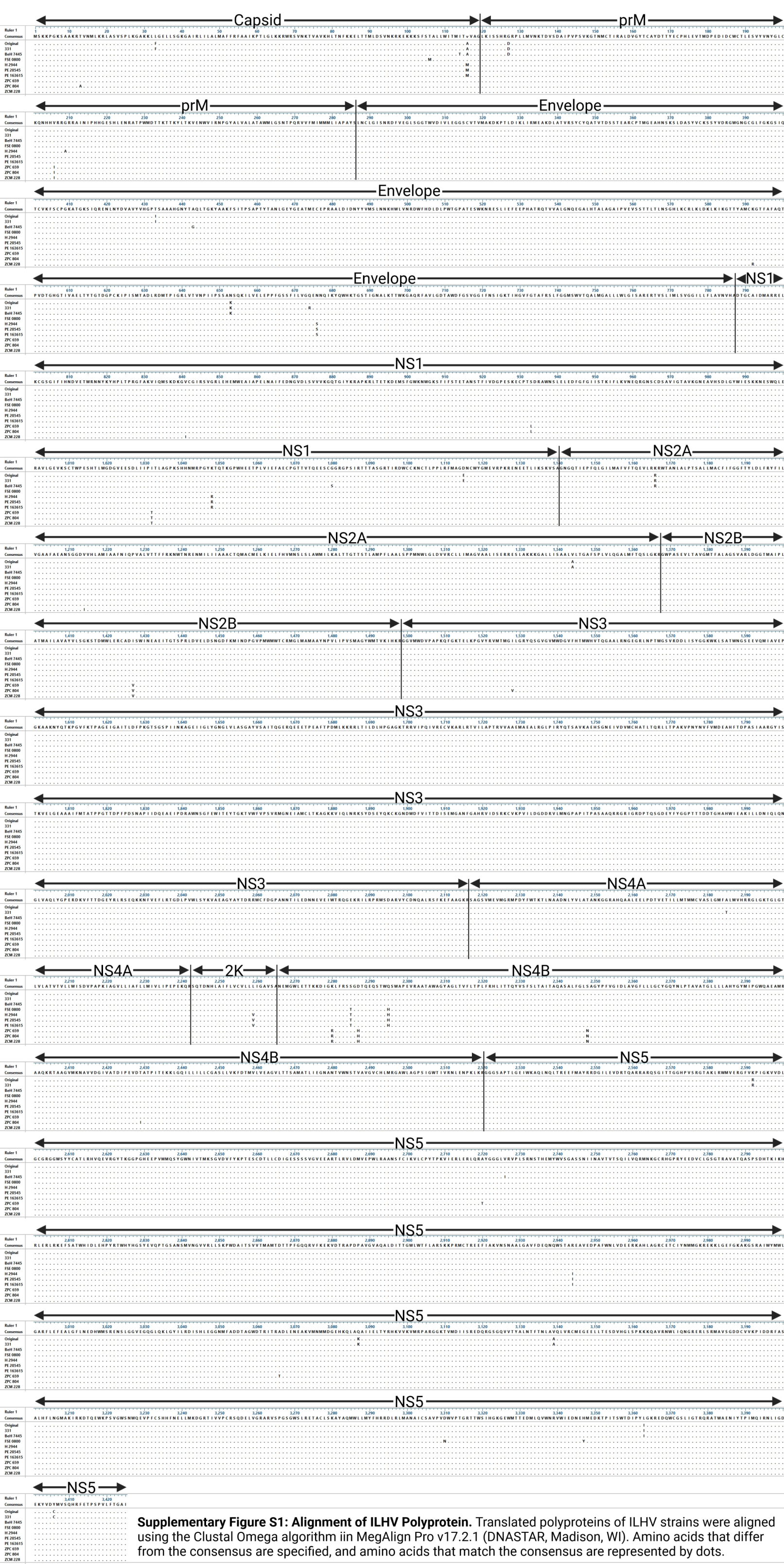

Supplement: Supplementary file 1 [file viruses-15-00195-s001.zip › Supplementary Figure S1 - ILHV Alignment.pdf]
